# Sharper, smaller, brighter: enhanced optical performance at the X06DA-PXIII beamline after the SLS 2.0 upgrade

**DOI:** 10.1107/S1600577526001992

**Published:** 2026-03-25

**Authors:** Benedikt Rösner, Vincent Olieric, Michael Boege, Rolf Follath, Sibylle Spielmann-Jäggi, Uwe Flechsig, Goran Lovric, Istvan Mohacsi, Katherine McAuley, Meitian Wang

**Affiliations:** a Center for Photon Science Paul Scherrer Institut Forschungsstrasse 111 5232Villigen PSI Switzerland; b Center for Accelerator Science and Engineering Paul Scherrer Institut Forschungsstrasse 111 5232Villigen PSI Switzerland; RIKEN SPring-8 Center, Japan

**Keywords:** SLS 2.0, beamline design, optics, hard X-ray, monochromator

## Abstract

The X06DA-PXIII beamline is a crystallography beamline on a superbend magnet at the Swiss Light Source. Since 2008, it has served the scientific community, contributing to 2650 Protein Data Bank structures as of today (*i.e.* before the SLS shutdown started in autumn 2023). Just before the upgrade of the storage ring, the beamline has been rebuilt with new X-ray optics to exploit the full potential of SLS 2.0. Its new design aims for a unique position in macromolecular crystallography beamlines worldwide: it exploits the excellent electron beam properties of small sizes below 10 µm, uses a toroidal mirror close to the source to harvest a large solid angle of the bending magnet beam, minimizes toroidal aberration with a horizontal 1:2 focusing concept and finally utilizes a high-quality Kirkpatrick–Baez mirror system to focus the beam onto the sample. Herein, we describe the beamline’s optical performance during first commissioning and demonstrate how the reduced emittance of the new storage ring improves its focusing capabilities and overall efficiency.

## Abstract 2

The X06DA-PXIII beamline at the Swiss Light Source, operating since 2008 and contributing to over 2650 crystal structures, has been fully rebuilt to harness the reduced emittance and improved performance of the SLS 2.0 storage ring. Its new optical design, featuring a toroidal mirror close to the source, horizontal 1:2 focusing, and a Kirkpatrick–Baez mirror system, enables efficient photon collection and tight beam focusing and thus improves overall efficiency.

## 1. Introduction

The Swiss Light Source (SLS) hosts around 20 beamlines, of which three are dedicated to macromolecular crystallography (MX): X06SA-PXI, X10SA-PXII and X06DA-PXIII, with a combined energy range between 5.5 and 20 keV. The upgrade of the SLS to SLS 2.0 had a significant impact on these beamlines. It included an increase in the storage-ring energy from 2.4 to 2.7 GeV and a significantly lower horizontal emittance (Willmott *et al.*, 2021 ▸) as a result of replacing the triple-bend achromat with a seven-bend achromat. This upgrade brought the SLS 2.0 into the league of so-called fourth-generation storage rings (Shin, 2021 ▸; Tavares *et al.*, 2014 ▸; Liu *et al.*, 2014 ▸; Raimondi, 2016 ▸). The new synchrotron greatly benefits from an increase in brightness and brilliant flux compared with that at the former storage ring (between a factor of six and 100, depending on their energy range and source type), not only due to the reduced emittance by approximately a factor of 30 but also since the X06SA-PXI and X10SA-PXII beamlines get new insertion devices, whereas the X06DA-PXIII beamline is operated with a new superbend magnet.

While the optics concept of the X06SA-PXI and X10SA-PXII undulator beamlines is described in another paper (Roesner *et al.*, 2024 ▸), we explain here the implications of the SLS upgrade for X06DA-PXIII. Its design at SLS 2.0 fills a particular gap in MX beamlines worldwide (Liebschner *et al.*, 2016 ▸; Mazzorana *et al.*, 2020 ▸; Ursby *et al.*, 2020 ▸; Chen *et al.*, 2023 ▸; Warren *et al.*, 2024 ▸; Yamamoto & Kumasaka, 2025 ▸; Ralston *et al.*, 2025 ▸; Orlans *et al.*, 2025 ▸; Wang, 2025 ▸): it exploits the excellent electron beam properties of small sizes below 10 µm (σ_*x*_ = 5.3 µm, σ_*y*_ = 6.8 µm) (Braun *et al.*, 2021 ▸) and uses a toroidal mirror close to the source to harvest a large solid angle of the bending magnet beam. Its photon energy range reaches from 3.0 keV to 13 keV. The optical concept minimizes the toroidal aberration with horizontal 1:2 focusing (MacDowell *et al.*, 2004 ▸) while vertically collimating, and finally utilizes high-quality Kirkpatrick–Baez (KB) mirrors to focus the small source onto the sample. Especially unique is the possibility to go to very low photon energies with very small spot sizes [most MX beamlines worldwide cannot reach energies below 5–7 keV, and, if they do, their focus size is much larger – usually > 100 µm compared with ∼20 µm FWHM as shown below (Liebschner *et al.*, 2016 ▸; Mazzorana *et al.*, 2020 ▸)].

This paper describes the concept of the new X06DA-PXIII beamline with the principal ideas for its optical design. Furthermore, we report on the optical performance observed during the first commissioning phase and examine how the reduced emittance of the new storage ring affects the beamline’s focusing properties. This is done by comparing the commissioning results obtained with the upgraded storage ring in early 2025 with those from the initial commissioning run using the new X-ray optics at the old SLS storage ring in fall 2023.

## 2. Concept for the PXIII beamline at SLS 2.0

### 2.1. Optical design

The optical design of the upgraded X06DA-PXIII is based on the simple and reliable design of the former X06DA-PXIII beamline. The original setup consisted of a flat, bendable collimating mirror reflecting downwards, followed by a double channel-cut monochromator and a bendable sagittal cylinder that deflected upwards and focused the beam onto the sample position. Owing to the relatively high horizontal emittance of the original SLS storage ring, achieving a tighter horizontal focus was limited by fundamental optical constraints. As a result, focusing with a toroidal mirror was sufficient despite its aberrations (MacDowell *et al.*, 2004 ▸).

**Figure 1:**
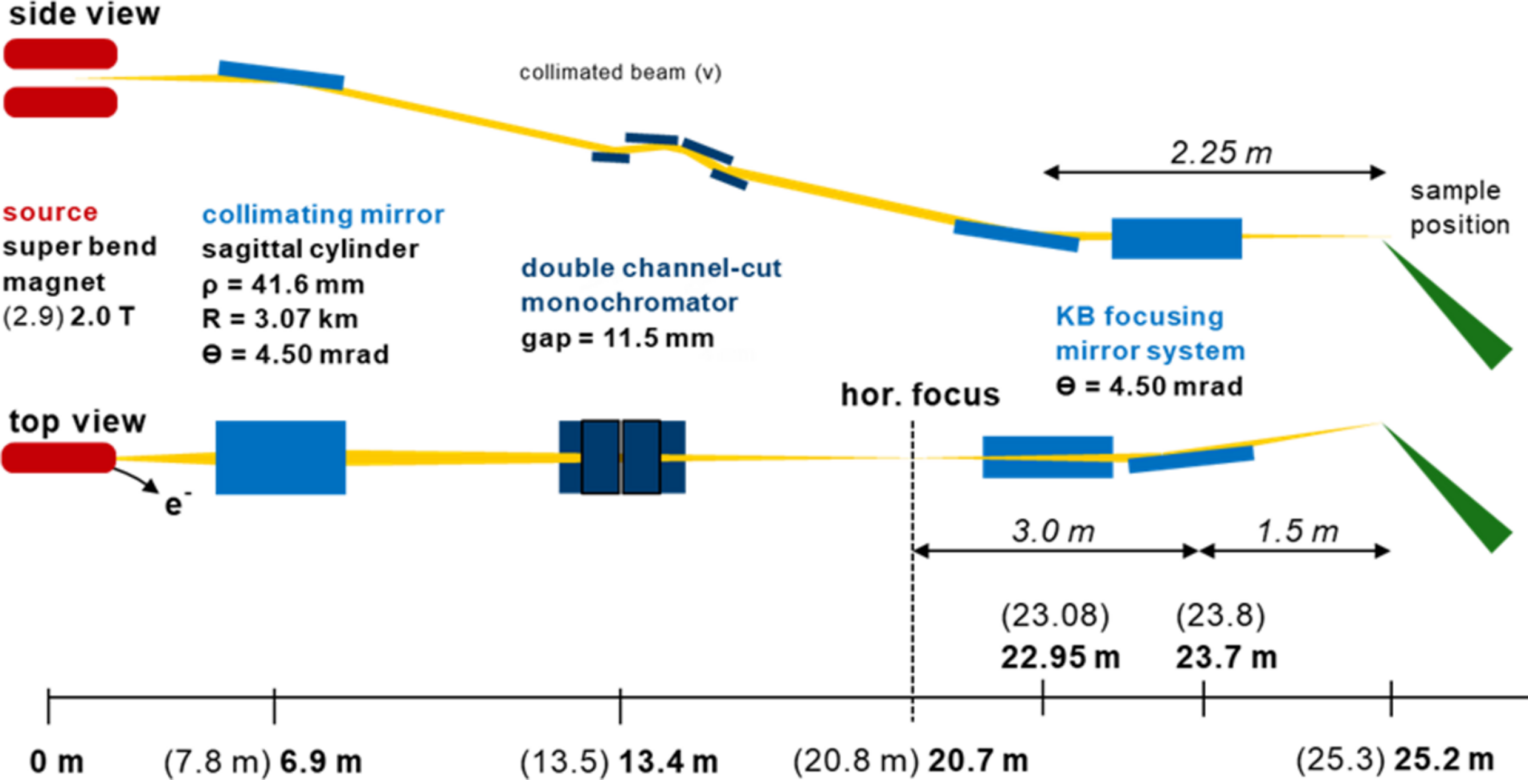
The new optical design of the upgraded X06DA-PXIII beamline. The optical components of the beamline consist of a collimating mirror located 6.9 m from the source, a double channel-cut monochromator at 13.4 m, a secondary source equipped with slits for the horizontal direction at 20.7 m, and a KB mirror system with a vertical working distance of 2.25 m and a horizontal working distance of 1.50 m. The focus point at the sample position is 25.2 m from the source point. The numbers in brackets refer to the values before the upgrade of the storage ring (SLS 1.0).

At the new storage ring of SLS 2.0, the horizontal emittance is lower by a factor of 30 (Braun *et al.*, 2021 ▸). This enables us to achieve a much smaller horizontal focus size, lowering it by an order of magnitude from 200 µm^2^ at the former beamline to ∼20 µm^2^ full width at half-maximum (FWHM) at the new storage ring. To achieve a tighter focusing at the new beamline, a KB mirror system with a significantly smaller focal length was chosen. The collimating mirror was kept in a downward-deflection geometry to match the existing geometry of the beamline, as the double channel-cut monochromator was also kept in place. However, the horizontal opening angle of the bending magnet front-end and the position of the new KB system would result in a relatively large beam size. This would either limit the focus spot strongly by the use of an extremely long horizontally focusing mirror (*i.e.* more than 1 m long), or cause significant loss of geometrical acceptance. Consequently, a horizontal intermediate focus upstream of the KB system was necessary. For this reason, the collimating mirror was designed as a bendable sagittal cylinder that vertically collimates the beam while focusing it horizontally to an intermediate focus of 39 µm FWHM (see Fig. 1 ▸). A pair of slits at the horizontal focus helps to reduce broadening of the horizontal spot size at the sample position by aberrations from the collimating mirror system that occur at front-end opening angles above 1.2 mrad. At smaller beamline acceptance, the slits provide a geometrical transmission of > 90% when opened to values above 66 µm (4σ of the horizontal focus size). The design or modifications of the individual optics components are described in the following sections.

### 2.2. Optical components of the beamline

#### 2.2.1. Collimating mirror unit

The collimating mirror unit hosts a downward-reflecting, bendable sagittal cylinder that is 1.16 m long. The sagittal radius is designed to fulfil the focus criterion for the ideal 1:2 focusing ratio at 4.5 mrad incident angle (MacDowell *et al.*, 2004 ▸) to the intermediate horizontal focus at 20.7 m. The corresponding radius in the sagittal direction for its final position at SLS 2.0, which is 6.9 m away from the source point, is therefore 41.4 mm. After polishing, the mirror was measured with a sagittal radius of 41.6 mm. In the meridional direction, the mirror can be bent to a radius of 3.07 km to collimate the beam in the vertical direction. This is achieved with a bender that can adapt the radius with high accuracy.

#### 2.2.2. Double channel-cut monochromator

The previously existing X06DA-PXIII beamline at the SLS was equipped with a double channel-cut monochromator that was placed in a vertically collimated beam and provided an energy range from 5.5 to 17 keV. For the new optical design, the monochromator was upgraded to reach lower photon energies. This was achieved with an extension of the Bragg range to higher angles as well as the use of channel-cut crystals with an enhanced gap size of 11.5 mm (the former gap size was 8 mm). With the new front-end design allowing a larger horizontal opening angle and the increased ring energy after the SLS upgrade, the power load on the monochromator has doubled. Consequently, the cooling of the crystals has been optimized by improving the thermal insulation and the crystal mount. This new geometrical configuration provides a reachable energy range between 3.0 keV to 15 keV. Note that the practical upper limit for the photon energy of the beamline is approximately 13 keV, due to the critical energy of the rhodium-coated mirrors at an angle of incidence of 4.5 mrad.

#### 2.2.3. KB mirrors

As its major focusing element, the beamline is equipped with a bendable KB mirror system. Its design is based on the KB focusing mirror systems that are installed at the three Aramis beamlines at SwissFEL (Ingold *et al.*, 2016 ▸). The mirrors are arranged with the vertically focusing mirror on the upstream side, followed by a horizontally focusing mirror. The vertically focusing mirror (VFM) has two optical stripes: one rhodium-coated stripe and a stripe with bare silicon for high harmonic rejection. It is worth noting that it is overfilled at small photon energies, which can be avoided by vertical slit blades at the horizontal secondary source. The working distance of the system is 1500 mm measured from the centre of the horizontally focusing mirror and 2250 mm from the VFM centre. Their incidence angles are 4.5 mrad, so that the VFM reflects the beam parallel to the floor again. The characteristic properties of all optical elements are listed in Table 1 ▸.

### 2.3. Ray tracing and increase in optical performance

To have a look at the focusing capabilities of the new beamline, we conducted ray tracing simulations using two ray tracing tools for cross-comparison and validation of the results, *PHASE* (Bahrdt *et al.*, 2014 ▸) and *XRT* (Klementiev & Chernikov, 2014 ▸; Klementiev & Chernikov, 2023 ▸). For both approaches, we found similar focus sizes (*XRT* results in a 3% smaller source and consequently also focus size). In the following, we show the ray tracing results of *PHASE* including the measured slope errors for the X-ray mirrors. For this purpose, we used the optical parameters presented in Table 1 ▸, in addition to the source sizes that were calculated based on the properties of the respective lattice parameters of the old and new versions of the Swiss Light Source (SLS 1.0 and SLS 2.0).

**Figure 2:**
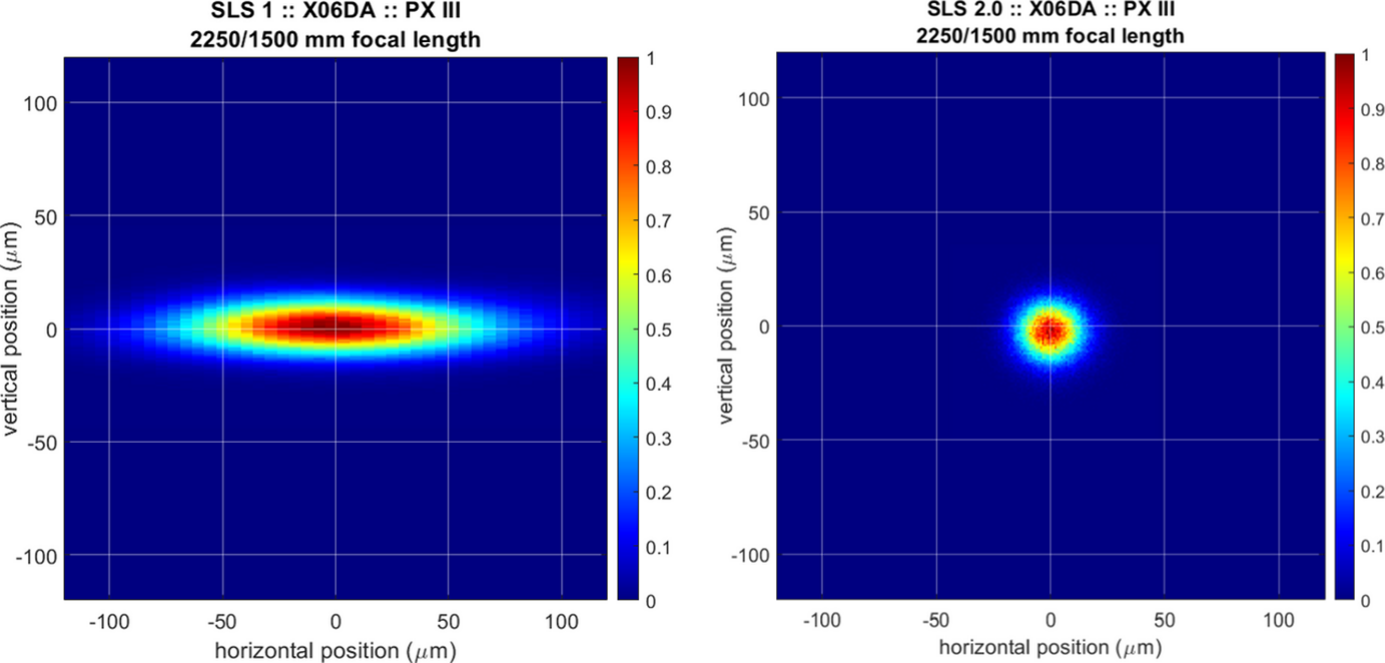
Ray tracing result from *PHASE* showing the focus of the PXIII beamline at 12.4 keV (left-hand side for SLS 1, right-hand side for SLS 2.0). The calculated focus sizes are 114 µm × 21 µm and 22 µm × 22 µm FWHM.

The simulated focus sizes before and after the SLS upgrade are shown in Fig. 2 ▸. Note that the focusing capabilities for the beamline are strongly limited by the aberrations of the collimating mirror system, especially when the horizontal front-end acceptance is increased, due to an over-illumination of the sagittal cylinder. For this purpose, we use a front-end opening angle of 0.8 mrad in the horizontal direction as reference, which also proved to be the limit in terms of heat load on the monochromator of the beamline at SLS 2.0. The expected focus sizes are 114 µm × 21 µm (horizontal × vertical) FWHM for the beamline at SLS 1.0, whereas they decrease to 22 µm × 22 µm FWHM at the upgraded storage ring (SLS 2.0).

### 2.4. Measured focus size

**Figure 3:**
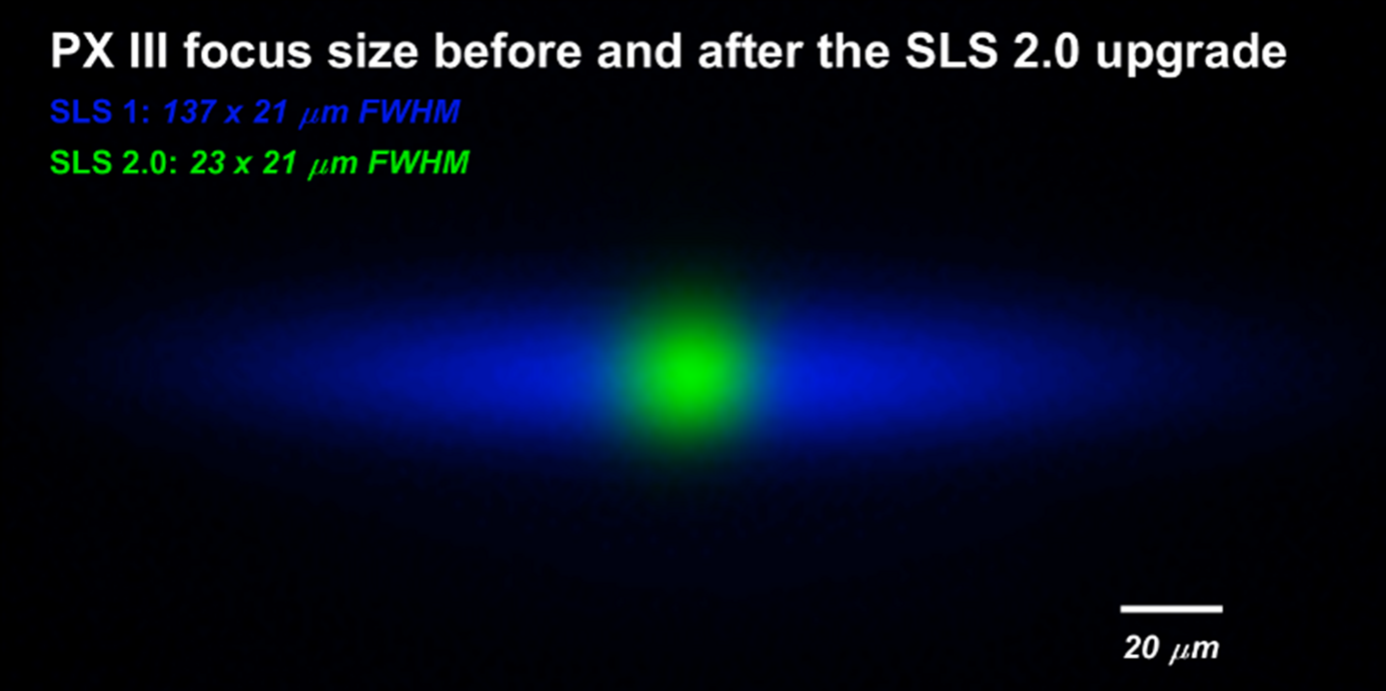
Overlay of the measured focus size of the X06DA-PXIII beamline at the sample position at SLS 1.0 (blue) and SLS 2.0 (green). The dimensions of the focus spots are 137 µm × 21 µm and 23 µm × 21 µm FWHM, respectively.

We measured the beam size at the focus plane during the initial operation at SLS 1.0 as well as after the SLS 2.0 upgrade. As a precondition, all optical elements were carefully adjusted to obtain the best possible focus. In the case of the collimating mirror, this task can be done with a screen at the horizontal focus. By correcting the mirror pitch (the incidence angle of the beam), the horizontal extension of the line focus can be adjusted. The bending of the collimating mirror was optimized until the beam was fully collimated, *i.e.* when the intensity on the screen at the horizontal intermediate focus changes homogeneously when detuning the second channel-cut crystal (if the beam is divergent or convergent, an intensity maximum can be observed travelling along the vertical dimension). Finally, the KB focusing mirror system was set to the pre-calibrated values for the ideal parabolic / elliptical shapes and fine-tuned until the spot size reached a minimum. The focus spot size was then determined with a fluorescent screen (yttrium aluminium garnet with a thickness of 100 µm, Fig. 3 ▸) and a calibrated optical microscope. The piezoelectric sample stage which usually holds the protein crystals for analysis was used as a reference to verify that the microscope’s calibration was correct.

During commissioning of the beamline at SLS 1.0 in autumn 2023, we determined a focus size of 137 µm × 21 µm FWHM, which is quite close to the expected values. In fact, while the vertical spot size is exactly as calculated, the horizontal dimension of the focus spot was 21% larger. After the upgrade of the storage ring to SLS 2.0, the observed spot size decreased to 23 µm × 21 µm FWHM.

## 3. Discussion

The measured values for the focus match well with the results from ray tracing simulations. In fact, the spot size after the SLS upgrade quantitatively matches the simulated values including the figure errors determined from the calibration measurements. The slightly larger horizontal beam size observed during SLS 1.0 operation cannot be attributed to the beamline layout, leaving deviations in the source size as the only explanation.

As SLS 1.0 consisted of electromagnets, the degrees of freedom for the electron beam orbit were much more flexible than after the upgrade, where the electromagnets were replaced with permanent magnets. As a consequence, obtaining knowledge of the exact source location at SLS 1.0 was much more difficult than after the storage ring upgrade. From electron beam dynamics settings, we estimate that the real X-ray source point of the X06DA-PXIII beamline was displaced by approximately 25 cm in the longitudinal direction from its nominal position. This displacement effectively introduces a larger apparent source size in the horizontal plane due to the projection angle of the optical axis of the beamline optics onto the electron orbit. As a result, even with ideal focusing conditions, the geometric image at the sample position reflects this increased effective horizontal source size.

The lower horizontal emittance and the improved brightness of the bending magnet source benefits the beamline’s performance. While the full quantitative advantage of emittance reduction is larger at insertion device beamlines, the performance increase at the X06DA-PXIII beamline is still remarkable. The calculated photon flux at SLS 1.0 including the geometrical acceptance of the beamline, the reflectivity of the mirrors and the bandwidth of the double channel-cut monochromator is 5.6 × 10^11^ photons s^−1^ (at a front-end opening of 1 mrad), the calculated flux at SLS 2.0 is 1.3 × 10^12^ photons s^−1^. At the sample position, with the measured spot sizes, we achieved a flux density of 1.7 × 10^14^ photons s^−1^ mm^−2^ at SLS 1.0, which increased now by a factor of 14 to 2.4 × 10^15^ photons s^−1^ mm^−2^. This increased usable flux leads to significantly higher throughput and productivity at X06DA-PXIII compared with before; a full crystallographic data set can now be taken in less than two minutes (including sample exchange).

## 4. Conclusions

After the first commissioning of X06DA-PXIII at SLS 2.0, we found that the beamline performance meets the simulated values precisely. The experimentally determined focus sizes after the SLS 2.0 upgrade agree with ray-tracing predictions, the measured 23 µm × 21 µm spot size quantitatively matches simulations that include the calibrated mirror figure errors. We found that the 137 µm × 21 µm horizontal focus at SLS 1.0 could not be fully explained by the design parameters of the storage ring or the beamline optics but rather attributed to a ∼ 25 cm longitudinal displacement of the true X-ray source point. Finally, while bending-magnet beamlines experience a more modest improvement due to larger effective emittance compared with insertion-device beamlines, the decreased horizontal emittance and enhanced brilliance at SLS 2.0 still result in a significant increase in flux density. Consequently, the upgrade at SLS 2.0 establishes the bending magnet as an attractive source, delivering improved performance for MX applications, particularly in autonomous, high-throughput, high-resolution experiments.

## Figures and Tables

**Table 1:** Characteristic properties of the optical elements The sample (focus) point of the beamline is at (25.40) 25.20 m. The numbers in brackets refer to the situation before the storage ring upgrade (SLS 1.0), whereas all other numbers are either not affected by the upgrade or represent the final situation at SLS 2.0.

	Collimating mirror	Channel-cut crystals	Intermediate focus (horizontal secondary source)	Vertically focusing KB mirror	Horizontally focusing KB mirror
Shape	Sagittal cylinder, bent to toroid	11.5 mm gap	Horizontal and vertical slits†	Flat, bent to plane elliptical	Flat, bent to plane elliptical
Position (m)	(7.65) 6.90	(13.54) 13.4	(20.84) 20.70	(23.08) 22.95	(23.84) 23.70
Source distance (m)	(7.65) 6.90	–	–	Infinity	3.00
Image distance (m)	13.80 (horizontal) / infinity (vertical)	–	–	2.25	1.50‡
Geometrical surface size (mm)	1160 × 70	40 × 40 (surface 1)	–	650	650
		100 × 40 (surface 2)			
Optical surface size (mm)	900 × 30	25 × 20	–	580	580
		90 × 20			
Footprint (4σ) at 12.4 keV (mm)	(876) 720	17.9	–	(672) 600	(470) 392
Bulk material	Si	Si (100)	–	Si	Si
Coating (30 nm)	Rh	None	–	None / Rh	Rh
Incidence angle (mrad) (if not °)	(4.27) 4.50	7.6°–41.2°	–	4.50	4.50
		9.2° @ 12.4 keV			
Tangential radius (km)	(3.64) 3.07	–	–	–	–
Sagittal radius (mm)	41.6	–	–	–	–
Tangential slope error (µrad) r.m.s.	0.87	–	–	< 1.55§	< 1.34§
Sagittal slope error (µrad) r.m.s.	9.07	–	–	–	–
Surface roughness (nm) r.m.s.	0.18	25	–	0.16	0.20
Cooling	Side-cooled with water	Indirectly cooled with a cold finger	–	–	–

† Can be used to prevent overfilling of the KB system.

‡ Focus position at 25.20 m.

§ In mounted configuration.
